# Prognostic Importance of Dyspnea for Cardiovascular Outcomes and Mortality in Persons without Prevalent Cardiopulmonary Disease: The Atherosclerosis Risk in Communities Study

**DOI:** 10.1371/journal.pone.0165111

**Published:** 2016-10-25

**Authors:** Mario Santos, Dalane W. Kitzman, Kunihiro Matsushita, Laura Loehr, Carla A. Sueta, Amil M. Shah

**Affiliations:** 1 Division of Cardiovascular Medicine, Brigham and Women’s Hospital, Boston, MA, United States of America; 2 Department of Physiology and Cardiothoracic Surgery, Cardiovascular R&D Unit, Faculty of Medicine, University of Porto, Porto, Portugal; 3 Wake Forest Baptist Medical Center, Winston Salem, NC, United States of America; 4 Johns Hopkins Bloomberg School of Public Health, Baltimore, MD, United States of America; 5 University of North Carolina, Chapel Hill, NC, United States of America; University of Tampere, FINLAND

## Abstract

**Background:**

The relationship between dyspnea and incident heart failure (HF) and myocardial infarction (MI) among patients without previously diagnosed cardiopulmonary disease is unclear. We studied the prognostic relevance of self-reported dyspnea for cardiovascular outcomes and all-cause mortality in persons without previously diagnosed cardiopulmonary disease.

**Methods and Results:**

We studied 10 881 community-dwelling participants (mean age 57±6, 56% women, 25% black) who were free of prevalent cardiopulmonary disease from Atherosclerosis Risk in Communities Study. Dyspnea status at study entry using the modified Medical Research Council (mMRC) scale. The primary outcomes were time to HF, MI or all-cause death. Dyspnea prevalence was 22%, and was mild (mMRC grade 1 or 2) in 21% and moderate-to-severe (mMRC 3 or 4) in 1%. The main correlates of dyspnea were older age, female sex, higher BMI and active smoking. Over a follow-up of 19±5 years, greater self-reported dyspnea severity was associated with worse prognosis. Mild dyspnea was associated with significantly heightened risk of HF (adjusted Hazard Ratio, HR,1.30; 95% CI: 1.16–1.46), MI (adjusted HR 1.34; 95%CI: 1.20–1.50), and death (adjusted HR 1.16; 95%CI: 1.06–1.26), with moderate/severe dyspnea associated with an even greater risk (adjusted HR 2.14, 95%CI: 1.59–2.89; 1.93, 95%CI: 1.41–2.56; 1.96, 95%CI: 1.55–2.48, respectively).

**Conclusion:**

In community-dwelling persons free of previously diagnosed cardiopulmonary disease, self-reported dyspnea is common and, even when of mild intensity, it is independently associated with a greater risk of incident HF, MI, and death. Our data emphasize the prognostic importance of even mild self-reported dyspnea for cardiovascular outcomes.

## Introduction

Dyspnea is a subjective experience of breathing discomfort that consists of distinct sensations of varying intensity[1]. In the general population, the overall prevalence of dyspnea is variable across studies,[2] related to differences in the distribution of known correlates of dyspnea such as age, gender, and smoking status, in the burden of comorbidities, and in the instrument used to measure dyspnea[3]. Despite variable prevalence estimates, dyspnea has been consistently associated with greater mortality in the general population[4, 5]. It is a more powerful predictor of clinical outcomes than objective physiologic measures such as pulmonary function testing[6, 7] in the general population, or angina in patients referred for cardiac evaluation[8]. However, little data is available regarding the prognostic relevance of self-reported dyspnea for non-fatal cardiovascular (CV) outcomes, such as incident myocardial infarction (MI) or heart failure (HF). In addition, scarce data is available regarding the prevalence and prognostic significance of dyspnea in African Americans, a population that carries a sizable proportion of the cardiovascular disease burden[9, 10].

We determined the prognostic relevance of self-reported dyspnea for CV outcomes in persons without previously diagnosed cardiopulmonary disease. We examined the correlates of self-reported dyspnea in a large biracial community-based cohort. We then defined the relationship between the presence and severity of dyspnea and all-cause mortality among participants with prevalent CV disease (MI, HF, stroke), pulmonary disease (COPD, asthma), or neither. Finally, among participants without previously diagnosed cardiopulmonary disease, we determined the prognostic relevance of dyspnea for incident MI and HF.

## Methods

### Study population

The Atherosclerosis Risk in Communities (ARIC) Study is an ongoing, prospective observational study. Detailed study rationale, design, and procedures have been previously published[11]. The original cohort included 15 792 persons aged 45 to 64 years recruited between 1987 and 1989 (Visit 1) from four communities in the United States: Forsyth County, North Carolina; Jackson, Mississippi; Minneapolis, Minnesota; and Washington County, Maryland. Three subsequent follow-up visits occurred at 3-year intervals, with annual telephone interviews conducted between visits and currently ongoing. The ARIC study has been approved by Institutional Review Boards (IRB) at all participating institutions: University of North Carolina at Chapel Hill IRB, Johns Hopkins University IRB, University of Minnesota IRB, and University of Mississippi Medical Center IRB. Study participants provided written informed consent at all study visits.

This analysis was restricted to ARIC participants who attended Visit 2 (1990–1992) and completed the dyspnea questionnaire (n = 13,425). We restricted our analyses to self-described black or white participants (40 exclusions due to other race). We analyzed a total of 13 385 U.S. adults aged 46 to 70 year-old followed prospectively through 2011 (median follow-up of 19±5 years). From among this population, we identified participants free of prevalent cardiovascular and pulmonary disease (n = 10 881), defined as the absence of a previous history of HF, MI, stroke, COPD, and asthma.

### Dyspnea Assessment

The modified British Medical Research Council (mMRC) instrument is an activity-based dyspnea scale that describes five grades of dyspnea according to a positive response to the following questions (from the original MRC questionnaire[12]): dyspnea only with strenuous exercise (grade 0 or normal); dyspnea when hurrying on level ground or up a slight hill (grade 1); dyspnea when walking at one’s own pace on level ground (grade 2); dyspnea when walking 100 yards or for a few minutes (grade 3); and dyspnea at rest (grade 4). This questionnaire was administered to ARIC participants at Visit 2. We further arbitrarily grouped subjects as having no dyspnea (mMRC grade 0), mild dyspnea (mMRC grade 1 or 2), and moderate to severe dyspnea (mMRC grade 3 or 4).

### Covariates

Established definitions for hypertension, obesity, diabetes mellitus, HF, coronary heart disease (CHD), stroke, and smoking status were used as previously described in the ARIC study[13]. Briefly, sitting BP was measured. Recent use of antihypertensive medications, and smoking status were self-reported. Fasting plasma total cholesterol and Serum glucose were measured. Body mass index (kg/m2) was computed from measured weight and height. Preexisting heart failure was defined as: (1) self report of recent medications for heart failure, or (2) Stage 3 or “manifest heart failure” by Gothenburg criteria which rest on a physician’s judgment based on physical exam and history. Preexisting coronary heart disease at baseline was defined by self-reported prior physician diagnosis, or by evidence of MI by 12-lead ECG. Preexisting stroke was defined by any self-reported prior physician diagnosis of stroke. Self-reported COPD and asthma were assessed by the answer to the question “Has your doctor ever said you had: 1) chronic lung disease, such as chronic bronchitis, or emphysema; 2) asthma?”. Physical activity was expressed as the product of average metabolic equivalents by minutes during a week (METs*min/week) of moderate-to-vigorous activity. Estimated glomerular filtration rate (eGFR) was calculated using the CKD Epidemiology Collaboration (CKD-EPI) equation[14]. Medication history was obtained at Visit 2 by self-report of medication use during the previous two weeks and by reviewing medications brought by the participants to their visit. All covariates were ascertained at Visit 2 except for physical activity which was ascertained at Visit 1.

### Clinical Events

ARIC participants undergo surveillance for incident CHD events (fatal CHD, definite or probable MI, or coronary revascularization) and all-cause mortality as previously described in detail[15]. Briefly, incident CHD events were defined as the first occurrence of a fatal or non-fatal MI ascertained through surveillance, abstraction, and physician adjudication of hospitalizations with CHD-related ICD codes[15] and annual participant contact. Incident HF was based on HF hospitalization or HF death according to ICD codes (code 410 in any position) obtained by ARIC surveillance of hospital discharges[16]. Deaths were ascertained using National Death Index.

### Statistical analysis

Summary statistics were calculated as counts and percentages for categorical data and means and standard deviation for continuous data, by dyspnea category. Comparisons of baseline characteristics between groups were made using Pearson Chi-squared test and analysis of variance (ANOVA). To determine the cross-sectional correlates of dyspnea, we used linear regression (mMRC scale as dependent variable) and logistic regression (moderate-to-severe dyspnea as dependent variable); only covariates with a p<0.05 in univariate analysis were included in the multivariate model. We determined the rates of mortality, incident HF, and incident MI per 100 person-years at risk, using the ARIC Visit 2 date as baseline and stratified by dyspnea category. We used multivariable Cox proportional hazards regression models to assess the unadjusted and adjusted association of dyspnea with mortality, incident HF, incident MI, or the composite of these. Minimally adjusted models adjusted for age, sex, race, and Center while fully adjusted models also adjusted for race, hypertension, diabetes, body mass index (BMI), current and former smoking status, systolic blood pressure, heart rate, eGFR, hemoglobin, left ventricular hypertrophy by ECG, statin use, antihypertensive medication use, anticoagulant use and aspirin use at baseline (Visit 2), as well as physical activity measure at Visit 1[17]. Covariates included in the full multivariable models were selected using a forward stepwise selection procedure (retention at p<0.05) with all variables that varied significantly between dyspnea categories (Table 1) included as candidate covariates. The proportional hazards assumption was assessed by visual inspection of Schoenfeld residuals. We additionally performed subgroup analyses by age, sex, race, and obesity status.

**Table 1:** Baseline characteristics of the studied cohort, by the presence of dyspnea.

Characteristic	Overall	Non-dyspnea	Mild dyspnea	Moderate-to-severe dyspnea	P-value
	(N = 10 881)	(n = 8 463)	(n = 2 270)	(n = 148)	
Demographic					
Age, y	57±6	56±6	58±6	57±6	<0.001
Male gender, n (%)	4781 (44)	4 014 (47)	728 (32)	39 (26)	<0.001
Race, n (%)					
White	8 157 (75)	6 312 (75)	1 793 (79)	52 (35)	<0.001
Black	2 724 (25)	2 151 (25)	477 (21)	96 (65)	
Anthropometric & ECG					
BMI, Kg/m^2^	27.7±5.2	27.3±4.8	29.2±5.9	32.5±7.8	<0.001
SBP, mmHg	121±19	120±18	123±19	129±25	<0.001
DBP, mmHg	72±10	72±10	72±10	75±12	0.002
HR, bpm	65±10	65±10	67±11	68±11	<0.001
LVH, n (%)	240 (2)	169 (2)	55 (2)	16 (11)	<0.001
LBBB, n (%)	9 (0)	6 (0)	2 (0)	1 (1)	0.04
Other than Sinus Rhythm, n (%)	31 (0)	22 (0)	9 (1)	0 (0)	0.45
Risk factors & Comorbidities					
Hypertension, n (%)	3 602 (33)	2 580 (28)	939 (42)	83 (57)	<0.001
Diabetes, n (%)	736 (7)	504 (6)	204 (9)	28 (19)	<0.001
Dyslipidemia, n (%)	3 185 (30)	2 352 (28)	784 (35)	49 (34)	<0.001
Obesity, n (%)	2 977 (27)	2 014 (24)	875 (39)	88 (60)	<0.001
Smoking status					
Current, n (%)	2 335 (22)	1 655 (20)	641 (28)	39 (26)	<0.001
Former, n (%)	3 995 (37)	3 195 (38)	749 (33)	51 (35)	0.001
Alcohol daily intake, n (%)	3 844 (35)	3 122 (37)	695 (31)	27 (18)	<0.001
Alcohol daily intake, g/week	38±93	38±90	37±102	19±58	0.04
Physical Activity, METs*min/week	623±766	672±795	461±630	266±498	<0.001
Blood Analysis and Lung Function					
FEV1/FVC, %	77±4	77±4	77±4	73±5	<0.001
FEV1/FVC < 70%, n (%)	1 493 (14)	1 142 (14)	288 (13)	63 (43)	<0.001
Hemoglobin, g/dL	13.7±1.3	13.7±1.3	13.6±1.3	13.3±1.5	<0.001
eGFR, mL/min/m2	65±12	65±11	63±12	65±15	<0.001
CKD, n (%)	3 813 (35)	3 333 (34)	927 (41)	53 (36)	<0.001
Medication					
Anti-hypertensive, n (%)	2 984 (27)	2 120 (25)	789 (35)	75 (51)	<0.001
Statin, n (%)	216 (2)	164 (2)	45 (2)	7 (5)	0.05
Anticoagulant, n (%)	31 (0)	18 (0)	12 (1)	1 (1)	0.03
Aspirin, n (%)	5 207 (48)	3 901 (46)	1 233 (55)	73 (50)	<0.001

Abbreviations: BMI, body mass index; CKD, chronic kidney disease (eGFR < 60 mL/min/1.73m^2^); DBP, diastolic blood pressure; ECG, electrocardiogram; eGFR, estimated glomerular filtration rate; FEV1, forced expiratory volume in the first second; FVC, functional vital capacity; HR, heart rate; LBBB, left bundle branch block; LVH, left ventricular hypertrophy; MET, metabolic equivalent; SBP, systolic blood pressure.

Two-sided p values of <0.05 were considered significant. Analyses were performed using Stata version 12.1 (Stata Corp., College Station, TX, USA).

## Results

### Population characteristics

**Fig 1:**
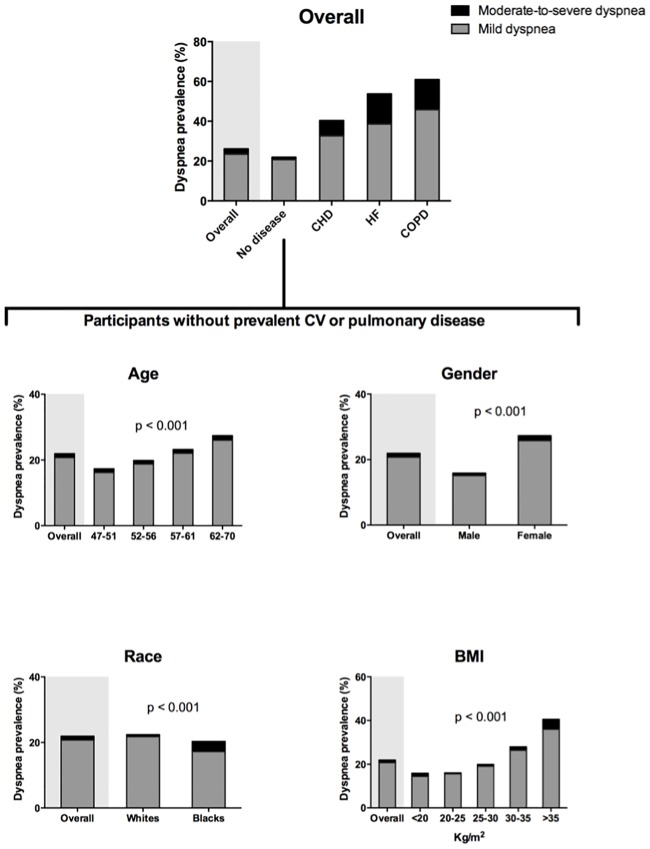
Dyspnea prevalence, by prevalence of cardiopulmonary disease, age, gender, race, and obesity.

Of the 13 385 participants included in this analysis, the mean age was 57±6 years, 55% were female, and 25% self-reported black ethnicity. CHD, HF and stroke prevalence were 6%, 4% and 1.5%, respectively. The prevalence of COPD and asthma were both 5%. The overall prevalence of dyspnea was 26%, with mild dyspnea accounting for the majority (23%), and only 3% reporting moderate-to-severe dyspnea (Fig 1). Among the 10 881 participants without previously diagnosed CV or pulmonary disease, dyspnea prevalence was similar at 22% (21% mild, 1% moderate-to-severe; Table 1). Women had a higher prevalence of dyspnea than men (27% vs 16%, p<0.001). White participants had similar prevalence of dyspnea to black participants (23% vs 21% respectively; p = 0.09), although the prevalence of moderate-to-severe dyspnea was greater in blacks than whites (4% vs 1% respectively; p<0.001).

### Participants with previously diagnosed cardiopulmonary disease

**Fig 2:**
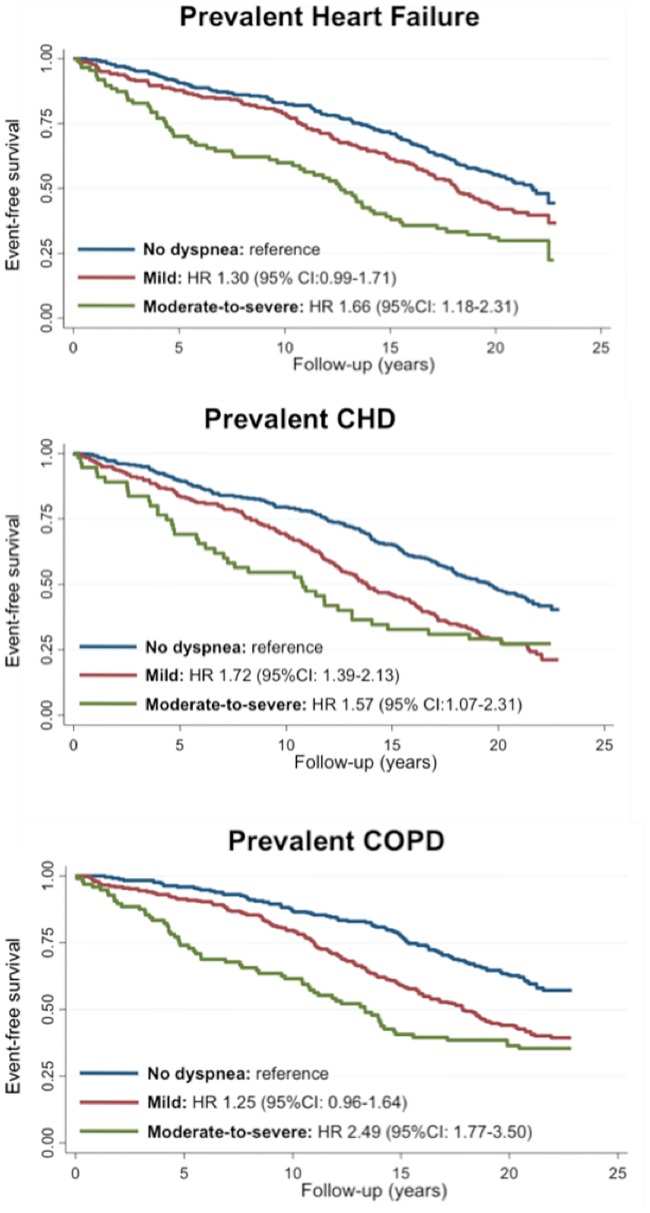
Mortality according to dyspnea severity among patients with (A) prevalent heart failure (n = 575), (B) prevalent coronary heart disease (N = 721), and (C) prevalent chronic obstructive pulmonary disease (n = 630).

Prevalent CHD, HF, and COPD were all independently associated with greater odds of any dyspnea self-report (OR 2.40 [2.04–2.84]; OR 2.74 [2.29–3.28]; OR 4.51 [3.79–5.37], respectively), after multivariable adjustment. They were also independently associated with greater odds of participant report of moderate-to-severe dyspnea (OR 4.35 [3.15–6.00]; 5.92 [4.51–7.77]; 9.64 [7.35–12.62], respectively). The presence of any self-reported dyspnea was associated with heightened risk of dying in participants with prevalent CHD, HF and COPD (Table 2), with a greater risk in those with moderate-to-severe dyspnea (Fig 2).

**Table 2:** Multivariable adjusted hazard ratios for death and incident heart failure and myocardial infarction with dyspnea.

	Number of events/ Total at risk	Event Rate Per 100 person-years (95% CI)	Unadjusted HR (95% CI)	Adjusted HR (95% CI)
**Men**				
**Incident HF**				
No dyspnea	571/4 014	0.79 (0.73–0.86)	Reference	Reference
Any dyspnea	191/767	1.58 (1.37–1.82)	1.89 (1.61–2.23)	1.34 (1.12–1.59)
**Incident CHD**				
No dyspnea	517/4 014	0.72 (0.66–0.79)	Reference	Reference
Any dyspnea	153/767	1.27 (1.08–1.49)	1.66 (1.38–1.99)	1.29 (1.06–1.57)
**Death**				
No dyspnea	1239/4 014	1.62 (1.53–1.72)	Reference	Reference
Any dyspnea	382/767	2.89 (2.61–3.20)	1.68 (1.50–1.88)	1.33 (1.17–1.50)
**Women**				
**Incident HF**				
No dyspnea	482/4 014	0.57 (0.52–0.62)	Reference	Reference
Any dyspnea	334/767	1.12 (1.01–1.26)	1.92 (1.67–2.21)	1.38 (1.19–1.60)
**Incident CHD**				
No dyspnea	292/4 014	0.34 (0.31–0.38)	Reference	Reference
Any dyspnea	166/767	0.55 (0.47–0.64)	1.55 (1.28–1.88)	1.22 (1.00–1.50)
**Death**				
No dyspnea	917/4 449	1.04 (0.97–1.10)	Reference	Reference
Any dyspnea	474/1651	1.49 (1.37–1.63)	1.32 (1.18–1.47)	1.10 (0.98–1.24)

Abbreviations: Minimally adjusted HR included in the models the following covariates: age, gender, race and field center. Adjusted HR included in the models the following covariates: visit center, age, sex, race, hypertension, diabetes, body mass index (BMI), current and former smoking status, systolic blood pressure, heart rate, eGFR, hemoglobin, left ventricular hypertrophy, statin use, antihypertensive medication use, anticoagulant use and aspirin use at baseline and physical activity.

### Participants without previously diagnosed cardiopulmonary disease

#### Correlates of any dyspnea (mMRC>0)

Among participants without previously diagnosed CV or pulmonary disease, the prevalence of dyspnea was 22% (mild: 21%; moderate-to-severe: 1%). Demographic characteristics, co-morbidities, LVH, and pulmonary function were all associated with dyspnea self-report (S2 Table). In multivariable analysis, age, sex, race, BMI, diabetes, hypertension and active smoking status were independent predictors of dyspnea. Together, the variables associated with dyspnea self-report only explained 7% of the variance in self-report of any dyspnea in this cohort.

#### Correlates of moderate-to-severe dyspnea (mMRC 3 or 4)

Among participants without previously diagnosed CV and pulmonary disease, the prevalence of moderate-to-severe dyspnea was 1%. Cardiovascular risk factors such as BMI and diabetes were significantly associated with moderate-to-severe dyspnea, while smoking status and FEV1/FVC ratio were not (S2 Table). Female sex was also an independent predictor of moderate-to-severe dyspnea. Similar to dyspnea in the overall population, these variables together only explained 8% of the variance in self-report of moderate-to-severe dyspnea.

#### Prognostic significance of dyspnea in persons without prevalent cardiopulmonary disease: Mortality

Participants were followed from 1990–1992 through December 31, 2011, with a mean of 19±5 years of follow-up. All-cause mortality occurred in 32% (n = 4 240) of the overall cohort, 2 352 in men and 1 888 in women. Table 2 shows mortality rates according to the presence of dyspnea among participants with no prevalent CV and pulmonary disease. The median time to death was 14.7 [9.7–18.4] years. Dyspnea self-report was associated with a significant 20% higher risk of dying during the follow-up period (Hazard Ratio, HR, 1.20 [1.10–1.31]). Both mild dyspnea (HR 1.16 [1.06–1.26]) and moderate-to-severe dyspnea (HR 1.96 [1.55–2.48]) were independently associated with greater mortality when compared to no dyspnea, with a greater risk associated with moderate-to-severe dyspnea than mild dyspnea.

In the overall cohort (including those with and without previously diagnosed cardiopulmonary disease), dyspnea was associated with a 25% increase in the risk of dying (HR-1.25 95% CI: 1.16–1.34) after adjusting for the presence of cardiopulmonary disease. While dyspnea was a significant predictor of all-cause mortality both among persons with and without prevalent cardiopulmonary disease, the magnitude of risk associated with dyspnea was greater in those with previously known cardiopulmonary disease (HR 1.35, 95% CI 1.19–1.54 and HR 1.13, 95% CI 1.04–1.24 among those with and without prevalent cardiopulmonary disease respectively; p-value for interaction: <0.001).

#### Prognostic significance of dyspnea in persons without prevalent cardiopulmonary disease: Cardiovascular outcomes

**Fig 3:**
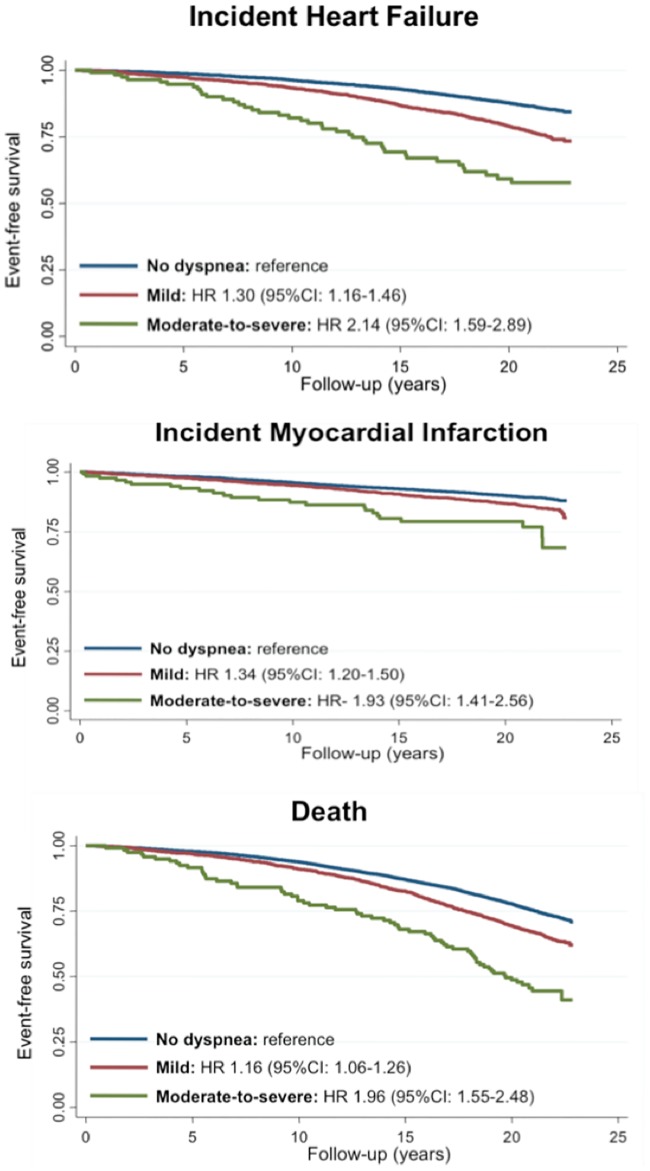
Survival curves of patients with no prevalent CV or pulmonary disease according to dyspnea status regarding death, incident HF and MI.

Incident myocardial infarction occurred in 1 128 (10%), and incident heart failure in 1 578 (15%) participants during follow-up. The median time to HF and MI were 13.8 [8.8–17.9] and 11.0 [6.3–16.2] years, respectively. Dyspnea was independently associated with higher risk of incident HF and of incident MI (Table 2; Fig 3). When compared to participants reporting no dyspnea, both mild dyspnea and moderate-to-severe dyspnea were each independently associated with a heightened risk of incident HF and of incident MI, with moderate-to-severe dyspnea conveying a greater risk than mild dyspnea (Table 2).

### Subgroup analyses among participants without prevalence CV or pulmonary disease

#### Women

The prevalence of both mild dyspnea and moderate-to-severe dyspnea was greater in women compared to men (25% vs 15%, p<0.001; 1.8% vs 0.8%, p<0.001, respectively). Men had higher mortality rate than women, and the risk of death associated with dyspnea self-report was also higher in men than women (hazard ratio: 1.33 [95% CI: 1.17–1.50] vs 1.10 [95% CI: 0.98–1.24]; p-value for interaction = 0.008; S3 Table). The risk of incident HF and incident MI associated with dyspnea self-report was similar among women compared to men, with no significant interaction between dyspnea self-report and sex for either of these outcomes.

#### Race

Blacks had a higher prevalence of cardiovascular comorbidities including hypertension, diabetes, obesity, and active smoking status than whites. Among both black and white participants, the presence of dyspnea was associated with a higher risk of incident HF, MI, and death compared to those with no dyspnea, without evidence of significant interaction between race and dyspnea (interaction p = 0.72, 0.31, 0.14 respectively; S4 Table). Of note, while the relative risk associated with dyspnea was similar between races, absolute mortality rates in blacks were significantly higher than in whites (1.20 vs 1.63 per 100-person-years), as were incident HF and MI rates (S4 Table), resulting in a greater increase in absolute event rate associated with dyspnea self-report in blacks.

#### Obesity

BMI was associated with a higher prevalence of dyspnea: Forty-one percent of participants with a BMI > 30 Kg/m^2^ reported dyspnea, compared to 20% of those with a BMI between 25–30 Kg/m^2^ (p<0.001). Among both obese and non-obese participants, self-reported dyspnea was associated with a heightened risk of outcomes without significant interaction between BMI and dyspnea in relation to risk of HF (p = 0.41), risk of MI (p = 0.14) and mortality (p = 0.52; S5 Table).

#### Age

Age was associated with a higher prevalence of dyspnea: 20% of participants in the lowest age quartile (mean age of 49 years) compared with 33% of participants in highest quartile (mean age of 65 years). No significant interaction between dyspnea self-report and age was found for incident HF (p = 0.65), or death (p = 0.51; S6 Table). The risk of incident MI was higher in older (>56 years; mean age: 62±3 years) compared to younger (<56 years; mean age: 52±3 years) participants (HR 1.04, 95% CI: 0.80–1.36 vs HR 1.34, 95% CI: 1.14–1.59, respectively; p for interaction = 0.007; S6 Table).

## Discussion

In this prospective study of over 13 000 adults from a community-based, biracial cohort, we observed an overall dyspnea prevalence of 26%. Older age, female sex, obesity, and active smoking status were the main correlates of dyspnea. Regardless of dyspnea severity or the presence of prevalent cardiopulmonary disease, self-report of dyspnea at study entry predicted death and non-fatal cardiovascular events. Notably, mild dyspnea in participants with no previous diagnosis of cardiopulmonary disease was consistently associated with a higher risk of incident HF, MI, and death. Despite being related to dyspnea, accounting for age, sex, race and obesity did not appreciably attenuate its overall prognostic significance.

A dyspnea prevalence of 26% in our middle-aged cohort is consistent with previous population-based reports[2] [4, 18]. Gronseth et al.[2] also used the mMRC scale to measure dyspnea in over 9000 subjects around the world and found an overall dyspnea prevalence of 27%. In the subset of US participants (416 subjects) the prevalence was much higher (47%), mainly due to self-reported mild dyspnea. The higher prevalence of obesity and heart disease in US subjects recruited into that study may contribute to the observed differences to our study. Importantly, in the ARIC cohort, the prevalence of self-reported dyspnea did not change much (26% to 22%) after excluding participants with a previous history of cardiovascular (CHD, HF, stroke) or pulmonary (COPD, asthma) disease. Consistent with previous studies [2, 19, 20], we found several correlates of dyspnea such as age, gender and obesity. Together these only explained 8% of the variance in dyspnea self-report. This may reflect unmeasured variables (e.g. cardiovascular function, musculoskeletal abnormalities) possibly causing dyspnea, individual and cultural differences in the recognition and expression of dyspnea, and limitations of the mMRC dyspnea measurement scale. This scale only measures the functional burden of dyspnea and does not capture other important dimensions like the sensorial and affective dimensions assessed by other measurement tools[1].

Dyspnea was an independent predictor of all-cause mortality. This association was observed for both mild and moderate-to-severe dyspnea, as well as for participants with and without history of cardiorespiratory diseases (COPD, asthma, HF, CHD, and stroke). Age, gender, and obesity correlated with self-reported dyspnea and, despite the mechanistic explanations that may link these features with dyspnea, they did not modify the prognostic impact of dyspnea on mortality and cardiovascular outcomes. Our finding that self-reported dyspnea predicts mortality is consistent with previous population-based studies looking both at healthy and diseased subjects[4, 8]. However, we extend existing knowledge on the prognostic value of dyspnea by demonstrating that—among participants with no previously diagnosed cardiovascular and respiratory disease—self-reported dyspnea is associated with a higher risk of developing HF or MI. With respect to magnitude of effect, a gradient of risk was noted from mild to moderate-to-severe dyspnea for incident HF, MI, and death.

There are several potential explanations for the observed association between self-reported dyspnea and non-fatal cardiovascular outcomes in participants without previously diagnosed disease. Although we had focused on participants without prevalent cardiopulmonary diseases, we cannot exclude the possibility of dyspnea being an expression of undiagnosed cardiovascular disease, even though the time lag between self-reported dyspnea and events (median of 11 to 13.8 years) may argue against it. Dyspnea may also be a surrogate marker of physical deconditioning due to low physical activity levels, which has been independently associated with cardiovascular outcomes[21]. Despite the adjustments for physical activity levels measured 2 years before dyspnea assessment, we cannot rule-out physical activity as a residual confounder or, alternatively, a mechanism through which dyspnea increase the risk of cardiovascular events. Finally, dyspnea may signal greater overall frailty, characterized by excess vulnerability to environmental stressors and reduced ability to maintain homeostasis after a destabilizing event[22].

Our data does not inform the causes and mechanisms behind dyspnea. Indeed traditional cardiac and pulmonary risk factors explained only a small amount of the variability in dyspnea self-report. However, this analysis clearly demonstrates that dyspnea is a common symptom in the general population, and—perhaps more importantly—an independent risk factor for adverse outcomes including incident HF and MI, and death. This holds true for middle-aged or elderly, women and men, and blacks or whites. Importantly, self-reported dyspnea (mild or moderate-to-severe) was also of similar prognostic value among obese or non-obese persons free of previously diagnosed cardiopulmonary disease. The 21% increase in risk of incident MI associated with dyspnea was significant, but more modest than other established coronary disease risk factors such a active smoking which was associated with 97% increase in risk in our study sample. Similarly, the significant 28% increase in risk for incident HF associated with dyspnea is more modest than the 55% increase in risk associated with obesity (defined as a BMI > 30Kg/m^2^; S7 Table). These consistent data stress the clinical importance of patient reported dyspnea, and emphasize the prognostic information that this subjective symptom conveys even in those patients with lower functional capacity, such as the elderly and obese. Further research is necessary to evaluate the mechanisms linking self-reported dyspnea to cardiovascular outcomes or the utility diagnostic testing to mitigate this risk. However, our findings suggest that providers should have a high index of suspicion for underlying cardiovascular—in addition to pulmonary—disease when confronted with patients reporting dyspnea, even in the context of advanced age or obesity.

Several limitations of our analysis should be considered. Given the observational nature of our study, we cannot rule-out potential confounding by unmeasured factors as contributors to the observed associations between dyspnea and cardiovascular outcomes. No echocardiographic data was available, therefore we cannot exclude the presence of undiagnosed structural heart disease (e.g. valvular heart disease). We used a single measurement of dyspnea, and therefore we cannot account for the influence of duration and variation in symptom reporting over time on the risk of developing cardiovascular events. Our variable of interest was a symptom that is commonly present in patients with HF and CHD. This could favor a detection bias if an uneven probability of undergoing diagnostic procedures occurred between participants reporting versus not reporting dyspnea. The consistent results regarding mortality, and the ascertainment of nonfatal cardiovascular outcomes using hospitalization, make this potential bias less important. The restricted number of blacks in the ARIC study increase the probability of a type II error as power was limited for examining race differences in the association between dyspnea and cardiovascular outcomes, although this remains a sizeable sample of black participants for an epidemiologic cohort study.

## Conclusions

Dyspnea is a prevalent symptom among middle-aged persons free of previously diagnosed cardiovascular disease and is independently associated with a heightened risk of developing heart failure, myocardial infarction and death. Regardless the underlying mechanism, dyspnea signals a worse clinical course independent of subject characteristics such as age, sex, race, or BMI.

## Supporting Information

S1 Table Baseline characteristics of the studied cohort, by the presence of cardiopulmonary disease. Abbreviations: BMI, body mass index; CKD, chronic kidney disease (eGFR < 60 mL/min/1.73m^2^); DBP, diastolic blood pressure; ECG, electrocardiogram; eGFR, estimated glomerular filtration rate; FEV1, forced expiratory volume in the first second; FVC, functional vital capacity; HR, heart rate; LBBB, left bundle branch block; LVH, left ventricular hypertrophy; MET, metabolic equivalent; SBP, systolic blood pressure.
(DOCX)

S2 Table Univariate and multivariate analysis of predictors of any dyspnea (grade 1–4 mMRC) and moderate-to-severe dyspnea (mMRC 3 or 4) of participants without prevalent cardiovascular and pulmonary disease. Abbreviations: BMI, body mass index; FEV1, forced expiratory volume in the first second; FVC, functional vital capacity; LVH, left ventricular hypertrophy.
(DOCX)

S3 Table Multivariable adjusted hazard ratios for incident death or cardiovascular events in participants without prevalent CV or pulmonary disease associated with dyspnea (any severity), by sex. P-values for interaction between dyspnea self-report and sex for outcomes: p = 0.558 for incident HF, p = 0.563 for myocardial infarction, and p = 0.008 for death. Abbreviations: Minimally adjusted HR included in the models the following covariates: age, gender, race and field center. Adjusted HR included in the models the following covariates: visit center, age, sex, race, hypertension, diabetes, body mass index (BMI), current and former smoking status, systolic blood pressure, heart rate, eGFR, hemoglobin, left ventricular hypertrophy, statin use, antihypertensive medication use, anticoagulant use and aspirin use at baseline and physical activity. CHD, coronary heart disease; CI, confidence interval; HF, heart failure; HR, hazard ratio.
(DOCX)

S4 Table Multivariable adjusted hazard ratios for incident death or cardiovascular events in participants without prevalent CV or pulmonary disease associated with dyspnea (any severity), by race. P-values for interaction between dyspnea self-report and race for outcomes: p = 0.718 for incident HF, p = 0.307 for myocardial infarction, and p = 0.138 for death. Abbreviations: Minimally adjusted HR included in the models the following covariates: age, gender, race and field center. Adjusted HR included in the models the following covariates: visit center, age, sex, race, hypertension, diabetes, body mass index (BMI), current and former smoking status, systolic blood pressure, heart rate, eGFR, hemoglobin, left ventricular hypertrophy, statin use, antihypertensive medication use, anticoagulant use and aspirin use at baseline and physical activity. CHD, coronary heart disease; CI, confidence interval; HF, heart failure; HR, hazard ratio.
(DOCX)

S5 Table Multivariable adjusted hazard ratios for incident death or cardiovascular events in participants without prevalent CV or pulmonary disease associated with dyspnea (any severity), by obesity status. P-values for interaction between dyspnea self-report and obesity for outcomes: p = 0.408 for incident HF, p = 0.136 for myocardial infarction, and p = 0.515 for death. Abbreviations: Minimally adjusted HR included in the models the following covariates: age, gender, race and field center. Adjusted HR included in the models the following covariates: visit center, age, sex, race, hypertension, diabetes, body mass index (BMI), current and former smoking status, systolic blood pressure, heart rate, eGFR, hemoglobin, left ventricular hypertrophy, statin use, antihypertensive medication use, anticoagulant use and aspirin use at baseline and physical activity. CHD, coronary heart disease; CI, confidence interval; HF, heart failure; HR, hazard ratio.
(DOCX)

S6 Table Multivariable adjusted hazard ratios for incident death or cardiovascular events in participants without prevalent CV or pulmonary disease associated with dyspnea (any severity), by age. P-values for interaction between dyspnea self-report and age for outcomes: p = 0.652 for incident HF, p = 0.007 for myocardial infarction, and p = 0.514 for death. Abbreviations: Minimally adjusted HR included in the models the following covariates: age, gender, race and field center. Adjusted HR included in the models the following covariates: visit center, age, sex, race, hypertension, diabetes, body mass index (BMI), current and former smoking status, systolic blood pressure, heart rate, eGFR, hemoglobin, left ventricular hypertrophy, statin use, antihypertensive medication use, anticoagulant use and aspirin use at baseline and physical activity. CHD, coronary heart disease; CI, confidence interval; HF, heart failure; HR, hazard ratio.
(DOCX)

S7 Table Effect estimates for all model covariates in full multivariable Cox proportional hazards models for death and incident cardiovascular events among subjects without previously known cardiopulmonary disease. Abbreviations: BMI, body mass index; eGFR, estimated glomerular filtration rate; FEV1, forced expiratory volume in the first second; FVC, functional vital capacity; HR, heart rate; LBBB, left bundle branch block; LVH, left ventricular hypertrophy; MET, metabolic equivalent; SBP, systolic blood pressure.
(DOCX)
