# Supplementary material for: Prognostic Importance of Dyspnea for Cardiovascular Outcomes and Mortality in Persons without Prevalent Cardiopulmonary Disease: The Atherosclerosis Risk in Communities Study
Source: PLoS One. 2016 Oct 25;11(10):e0165111. doi: 10.1371/journal.pone.0165111 (PMC5079579; doi:10.1371/journal.pone.0165111)
Supplement: S1 Table — Abbreviations: BMI, body mass index; CKD, chronic kidney disease (eGFR < 60 mL/min/1.73m2); DBP, diastolic blood pressure; ECG, electrocardiogram; eGFR, estimated glomerular filtration rate; FEV1, forced expiratory volume in the first second; FVC, functional vital capacity; HR, heart rate; LBBB, left bundle branch block; LVH, left ventricular hypertrophy; MET, metabolic equivalent; SBP, systolic blood pressure. (DOCX) [file pone.0165111.s001.docx]

**S1 Table**

| **Characteristic** | **Cardiopulmonary disease** | **No cardiopulmonary disease** | **P-value** |
| --- | --- | --- | --- |
|  |  |  |  |
|  | **(n=2504)** | **(n=10 881)** |  |
| Demographic |  |  |  |
| Age, y | 58±6 | 57±6 | <0.001 |
| Male gender, n (%) | 1257 (50) | 4781 (44) | <0.001 |
| Race, n (%) | 1 912 (76) | 8 157 (75) |  |
| White | 592 (24) | 2 724 (25) | 0.15 |
| Black |  |  |  |
| Anthropometric & ECG |  |  |  |
| BMI, Kg/m^2^ | 28.2±5.6 | 27.7±5.2 | <0.001 |
| SBP, mmHg | 123±19 | 121±19 | <0.001 |
| DBP, mmHg | 72±11 | 72±10 | 0.008 |
| HR, bpm | 67±11 | 65±10 | <0.001 |
| LVH, n (%) | 81 (3) | 240 (2) | 0.001 |
| LBBB, n (%) | 13 (1) | 9 (0) | <0.001 |
| Other than Sinus Rhythm, n (%) | 25 (1) | 31 (0) | <0.001 |
| Risk factors & Comorbidities |  |  |  |
| Hypertension, n (%) | 1212 (49) | 3 602 (33) | <0.001 |
| Diabetes, n (%) | 315 (13) | 736 (7) | <0.001 |
| Dyslipidemia, n (%) | 1005 (41) | 3 185 (30) | <0.001 |
| Obesity, n (%) | 762 (30) | 2 977 (27) | 0.002 |
| Smoking status |  |  |  |
| Current, n (%) | 652 (26) | 2 335 (22) | <0.001 |
| Former, n (%) | 1073 (43) | 3 995 (37) | <0.001 |
| Alcohol daily intake, n (%) | 775 (31) | 3 844 (35) | <0.001 |
| Alcohol daily intake, g/week | 35±96 | 38±93 | 0.24 |
| Physical Activity, METs*min/week | 599±761 | 623±766 | 0.16 |
| Blood Analysis and Lung Function |  |  |  |
| FEV1/FVC, % | 71±11 | 77±4 | <0.001 |
| FEV1/FVC < 70%, n (%) | 851 (34) | 1 493 (14) | <0.001 |
| Hemoglobin, g/dL | 13.9±1.4 | 13.7±1.3 | <0.001 |
| eGFR, mL/min/m2 | 64±13 | 65±12 | <0.001 |
| CKD, n (%) | 959 (38) | 3 813 (35) | 0.09 |
| Medication |  |  |  |
| Anti-hypertensive, n (%) | 1263 (50) | 2 984 (27) | <0.001 |
| Statin, n (%) | 102 (4) | 216 (2) | <0.001 |
| Anticoagulant, n (%) | 65 (3) | 31 (0) | <0.001 |
| Aspirin, n (%) | 1520 (61) | 5 207 (48) | <0.001 |
