# Supplementary material for: Prognostic Importance of Dyspnea for Cardiovascular Outcomes and Mortality in Persons without Prevalent Cardiopulmonary Disease: The Atherosclerosis Risk in Communities Study
Source: PLoS One. 2016 Oct 25;11(10):e0165111. doi: 10.1371/journal.pone.0165111 (PMC5079579; doi:10.1371/journal.pone.0165111)
Supplement: S2 Table — Abbreviations: BMI, body mass index; FEV1, forced expiratory volume in the first second; FVC, functional vital capacity; LVH, left ventricular hypertrophy. (DOCX) [file pone.0165111.s002.docx]

**S2 Table**

|  | | **Any dyspnea (N=10 733)** | | | | **Moderate-to-severe dyspnea (N=148)** | | | |
| --- | --- | --- | --- | --- | --- | --- | --- | --- | --- |
|  | | **Univariate** | | **Multivariate** | | **Univariate** | | **Multivariate** | |
| **Characteristic** | **N** | **OR (95% CI)** | **P-value** | **OR (95% CI)** | **P-value** | **OR (95% CI)** | **P-value** | **OR (95% CI)** | **P-value** |
| Age | 10 881 | 1.04 (1.03-1.05) | <0.001 | 1.05 (1.04-1.06) | <0.001 | 1.02 (0.99-1.06) | 0.20 |  |  |
| Male gender | 10 881 | 0.52 (0.47-0.57) | <0.001 | 0.46 (0.42-0.51) | <0.001 | 0.45 (0.31-0.65) | <0.001 | 0.58 (0.39-0.85) | 0.006 |
| Black | 10 881 | 0.91 (0.82-1.01) | 0.090 |  |  | 5.69 (4.05-8.00) | <0.001 | 1.92 (0.62-5.90) | 0.256 |
| BMI | 10 878 | 1.08 (1.07-1.09) | <0.001 | 1.09 (1.08-1.10) | <0.001 | 1.13 (1.10-1.15) | <0.001 | 1.09 (1.06-1.11) | <0.001 |
| Hypertension | 10 860 | 1.68 (1.53-1.84) | <0.001 | 1.42 (1.28-1.68) | <0.001 | 2.65 (1.91-3.68) | <0.001 | 1.35 (0.95-1.93) | 0.098 |
| Diabetes | 10 877 | 1.68 (1.43-1.97) | <0.001 | 1.3 (1.08-1.54) | 0.005 | 3.30 (2.17-5.02) | <0.001 | 1.63 (1.04-2.54) | 0.030 |
| Current Smoker | 10 879 | 1.61 (1.45-1.79) | <0.001 | 2.4 (2.09-2.68) | <0.001 | 1.31 (0.91-1.90) | 0.146 |  |  |
| Former Smoker | 10 879 | 0.82 (0.74-0.89) | <0.001 | 1.16 (1.04-1.30) | 0.01 | 0.91 (0.64-1.27) | 0.565 |  |  |
| LVH | 10 802 | 1.49 (1.12-1.97) | 0.006 | 1.24 (0.92-1.68) | 0.15 | 5.64 (3.30-9.64) | <0.001 | 2.88 (1.63-5.06) | <0.001 |
| FEV1/FVC | 10 881 | 1.0 (0.99-1.02) | 0.420 |  |  | 0.84 (0.81-0.87) | <0.001 | 0.94 (0.84-1.06) | 0.318 |
