# Supplementary material for: Prognostic Importance of Dyspnea for Cardiovascular Outcomes and Mortality in Persons without Prevalent Cardiopulmonary Disease: The Atherosclerosis Risk in Communities Study
Source: PLoS One. 2016 Oct 25;11(10):e0165111. doi: 10.1371/journal.pone.0165111 (PMC5079579; doi:10.1371/journal.pone.0165111)
Supplement: S6 Table — P-values for interaction between dyspnea self-report and age for outcomes: p = 0.652 for incident HF, p = 0.007 for myocardial infarction, and p = 0.514 for death. Abbreviations: Minimally adjusted HR included in the models the following covariates: age, gender, race and field center. Adjusted HR included in the models the following covariates: visit center, age, sex, race, hypertension, diabetes, body mass index (BMI), current and former smoking status, systolic blood pressure, heart rate, eGFR, hemoglobin, left ventricular hypertrophy, statin use, antihypertensive medication use, anticoagulant use and aspirin use at baseline and physical activity. CHD, coronary heart disease; CI, confidence interval; HF, heart failure; HR, hazard ratio. (DOCX) [file pone.0165111.s006.docx]

**S6 Table**

|  | **Number of events/ Total at risk** | **Event Rate Per 100 person-years (95% CI)** | **Unadjusted HR (95% CI)** | **Adjusted HR (95% CI)** |
| --- | --- | --- | --- | --- |
| **Below median (<56 years; mean 52±3 years)** | | | | |
| **Incident HF** |  |  |  |  |
| No dyspnea | 288/4 086 | 0.36 (0.32-0.41) | Reference | Reference |
| Any dyspnea | 144/939 | 0.82 (0.70-0.97) | 2.41 (1.97-2.96) | 1.60 (1.29-1.99) |
| **Incident CHD** |  |  |  |  |
| No dyspnea | 302/4 086 | 0.38 (0.34-0.43) | Reference | Reference |
| Any dyspnea | 80/939 | 0.45 (0.37-0.57) | 1.37 (1.06-1.76) | 1.04 (0.80-1.36) |
| **Death** |  |  |  |  |
| No dyspnea | 597/4 086 | 0.72 (0.66-0.78) | Reference | Reference |
| Any dyspnea | 197/939 | 1.05 (0.92-1.21) | 1.62 (1.37-1.91) | 1.23 (1.03-1.46) |
| **Above median (>56 years; mean 62±3 years)** | | | | |
| **Incident HF** |  |  |  |  |
| No dyspnea | 765/4 377 | 0.99 (0.92-1.06) | Reference | Reference |
| Any dyspnea | 381/1 479 | 1.57 (1.42-1.74) | 1.73 (1.53-1.97) | 1.28 (1.12-1.46) |
| **Incident CHD** |  |  |  |  |
| No dyspnea | 507/4 377 | 0.65 (0.60-0.71) | Reference | Reference |
| Any dyspnea | 239/1 479 | 0.97 (0.86-1.10) | 1.71 (1.47-2.00) | 1.34 (1.14-1.59) |
| **Death** |  |  |  |  |
| No dyspnea | 1 559/4 377 | 1.90 (1.81-2.00) | Reference | Reference |
| Any dyspnea | 659/1 479 | 2.55 (2.32-2.71) | 1.43 (1.31-1.58) | 1.19 (1.08-1.32) |
