# Supplementary material for: Prognostic Importance of Dyspnea for Cardiovascular Outcomes and Mortality in Persons without Prevalent Cardiopulmonary Disease: The Atherosclerosis Risk in Communities Study
Source: PLoS One. 2016 Oct 25;11(10):e0165111. doi: 10.1371/journal.pone.0165111 (PMC5079579; doi:10.1371/journal.pone.0165111)
Supplement: S7 Table — Abbreviations: BMI, body mass index; eGFR, estimated glomerular filtration rate; FEV1, forced expiratory volume in the first second; FVC, functional vital capacity; HR, heart rate; LBBB, left bundle branch block; LVH, left ventricular hypertrophy; MET, metabolic equivalent; SBP, systolic blood pressure. (DOCX) [file pone.0165111.s007.docx]

**S7 Table**

|  | **Death** | | **Incident MI** | | **Incident HF** | |
| --- | --- | --- | --- | --- | --- | --- |
|  | **HR (95%CI)** | **P-value** | **HR (95%CI)** | **P-value** | **HR (95%CI)** | **P-value** |
| Dyspnea | 1.13 (1.04-1.24) | 0.005 | 1.21 (1.05-1.40) | 0.008 | 1.28 (1.14-1.43) | <0.001 |
| Age, y | 1.10 (1.09-1.11) | <0.001 | 1.05 (1.03-1.06) | <0.001 | 1.09 (1.08-1.10) | <0.001 |
| Male gender | 1.71 (1.55-1.89) | <0.001 | 2.45 (2.08-2.88) | <0.001 | 1.66 (1.45-1.91) | <0.001 |
| Race (Black) | 1.07 (0.86-1.34) | 0.546 | 0.73 (0.48-1.10) | 0.131 | 1.20 (0.89-1.63) | 0.236 |
| BMI, Kg/m^2^ | 1.01 (1.01-1.02) | 0.001 | 1.00 (0.99-1.02) | 0.167 | 1.05 (1.04-1.06) | <0.001 |
| SBP, mmHg | 1.01 (1.00-1.01 | <0.001 | 1.01 (1.01-1.02) | <0.001 | 1.01 (1.01-1.02) | <0.001 |
| HR, bpm | 1.01 (1.01-1.02) | <0.001 | 1.01 (0.99-1.01) | 0.102 | 1.02 (1.01-1.02) | <0.001 |
| LVH | 1.48 (1.21-1.79) | <0.001 | 1.37 (1.00-1.87) | 0.049 | 1.70 (1.33-2.17) | <0.001 |
| Hypertension | 0.99 (0.88-1.14) | 0.975 | 0.81 (0.65-0.99) | 0.044 | 0.84 (0.70-1.00) | 0.053 |
| Diabetes | 1.71 (1.54-1.90) | <0.001 | 2.29 (1.95-2.68) | <0.001 | 2.28 (2.00-2.59) | <0.001 |
| Current smoker | 2.33 (2.10-2.58) | <0.001 | 1.97 (1.67-2.32) | <0.001 | 2.55 (2.21-2.94) | <0.001 |
| Former smoker | 1.15 (1.05-1.27) | 0.003 | 1.09 (0.94-1.26) | 0.275 | 1.33 (1.17-1.51) | <0.001 |
| Physical Activity, METs*min/week | 0.99 (0.99-0.99) | 0.003 | 0.99 (0.99-0.99) | 0.001 | 0.99 (0.99-1.00) | 0.050 |
| Hemoglobin | 0.95 (0.91-0.98) | 0.004 | 0.95 (0.90-1.01) | 0.094 | 0.92 (0.87-0.97) | 0.001 |
| FEV1/FVC, % | 0.98 (0.97-0.98) | <0.001 | 0.99 (0.99.1.01) | 0.617 | 0.98 (0.97-0.99) | <0.001 |
| eGFR, mL/min/1.73 m2 | 0.99 (0.99-1.00) | 0.080 | 0.99 (0.98-0.99) | <0.001 | 0.99 (0.98-0.99) | <0.001 |
| Antihypertensive | 1.20 (1.07-1.34) | 0.002 | 1.70 (1.41-2.05) | <0.001 | 1.76 (1.50-2.06) | <0.001 |
| Statin | 1.31 (1.04-1.65) | 0.024 | 1.42 (0.99-2.01) | 0.052 | 1.23 (0.90-1.68) | 0.201 |
| Anticoagulant | 3.26 (2.12-5.03) | <0.001 | 1.69 (0.70-4.10) | 0.243 | 3.08 (1.69-5.60) | <0.001 |
| Aspirin | 0.99 (0.92-1.07) | 0.844 | 1.01 (0.89-1.14) | 0.923 | 1.05 (0.95-1.16) | 0.376 |
